# Mammalian Metabolism of β-Carotene: Gaps in Knowledge

**DOI:** 10.3390/nu5124849

**Published:** 2013-11-27

**Authors:** Varsha Shete, Loredana Quadro

**Affiliations:** Department of Food Science and Rutgers Center for Lipid Research, Rutgers University, New Brunswick, NJ 08901, USA; E-Mail: varsha.shete@gmail.com

**Keywords:** β-carotene, β-carotene-15,15′-oxygenase, β-carotene-9′,10′-oxygenase, β-apocarotenoids, carotenoids, vitamin A, retinoids

## Abstract

β-carotene is the most abundant provitamin A carotenoid in human diet and tissues. It exerts a number of beneficial functions in mammals, including humans, owing to its ability to generate vitamin A as well as to emerging crucial signaling functions of its metabolites. Even though β-carotene is generally considered a safer form of vitamin A due to its highly regulated intestinal absorption, detrimental effects have also been ascribed to its intake, at least under specific circumstances. A better understanding of the metabolism of β-carotene is still needed to unequivocally discriminate the conditions under which it may exert beneficial or detrimental effects on human health and thus to enable the formulation of dietary recommendations adequate for different groups of individuals and populations worldwide. Here we provide a general overview of the metabolism of this vitamin A precursor in mammals with the aim of identifying the gaps in knowledge that call for immediate attention. We highlight the main questions that remain to be answered in regards to the cleavage, uptake, extracellular and intracellular transport of β-carotene as well as the interactions between the metabolism of β-carotene and that of other macronutrients such as lipids.

## 1. Introduction

Carotenoids are C_40_ tetraterpenoid pigments that are found in plants, fungi and bacteria. Mammals obtain carotenoids predominantly through foods of plant origin [1]. In plants, these compounds accumulate in the plastids giving the characteristic bright yellow, red and orange color to many fruits and vegetables [1]. In plants, they function as structural and functional accessories of the photosynthetic apparatus, specifically to serve as light-harvesting pigments and protect against photooxidative stress [2,3,4]. Plant carotenoids also function as precursors of various hormones and play a role in attracting pollinators and other agents that contribute to seed dispersal [5].

Decades of investigations have clearly shown that carotenoids obtained through the diet serve several beneficial functions in mammals, including humans, owing to their antioxidant properties, their ability to generate vitamin A as well as due to the emerging crucial signaling functions of their metabolites [6,7,8,9]. On the other hand, evidence for potential harmful actions of these compounds on human health also exists [10,11]. A complete understanding of the metabolism of these compounds is still needed to unequivocally determine the conditions under which these compounds may exert either beneficial or detrimental effects on human health. This knowledge will ultimately generate dietary recommendations adequate to different groups of individuals and populations worldwide.

The major emphasis of this review will be on β-carotene, the most abundant carotenoid found in human diet and tissues [12,13], even though references to other carotenoids important in human nutrition and health will be also made wherever appropriate. Although several studies have reported the beneficial effects of β-carotene due to its antioxidant properties [14,15,16], in this review we will address crucial functions performed by this carotenoid owing to its provitamin A activity. We will provide a general overview of the main aspects of β-carotene metabolism in mammals to highlight what we believe are the major questions still left to be answered in this field of research.

## 2. Carotenoid Classification

**Figure 1:**
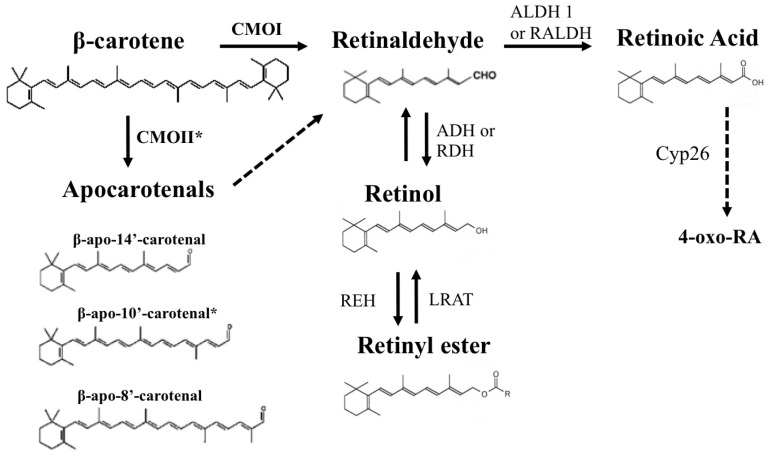
Summary of β-carotene metabolism. Symmetric oxidative cleavage of β-carotene at the 15,15′ double bond by the enzyme β-carotene-15,15′-oxygenase (CMOI or BCMO1 or BCO1) generates two molecules of retinaldehyde. Retinaldehyde can be oxidized into retinoic acid by members of the aldehyde dehydrogenase 1 family of enzymes (ALDH 1 or RALDH). Further oxidation of retinoic acid by enzymes that belong to the cytochrome P450 (CYP) 26 family converts retinoic acid into more polar compounds, including 4-oxo retinoic acid, which are believed to be transcriptionally inactive. Alternatively, different forms of alcohol dehydrogenase (ADH) from the MDR superfamily, and a variety of retinol dehydrogenases (RDH) from the SDR superfamily can reduce retinaldehyde to retinol, which can be further esterified into retinyl esters by the enzyme lecithin:retinol acyltransferase (LRAT). In addition, apocarotenals can be generated from β-carotene. The cleavage at the 9′,10′ double bond is catalyzed by β-carotene 9′,10′-oxygenase 2 (CMOII or BCDO2 or BCO2) and leads to the formation of β-apo-10′-carotenal (indicated by an asterisk) and β-ionone. Asymmetric cleavage at other double bonds may occur non-enzymatically or may be enzyme catalyzed. Some of the potential apocarotenals generated by asymmetric cleavage of β-carotene are depicted in the figure. The dashed arrow indicates that apocarotenals can be ultimately converted into one molecule of retinaldehyde. The mechanism of this conversion has not been completely elucidated. A chain shorthening mechanism has been proposed. However, recent reports from von Lintig’s and Harrion’s groups suggested that apocarotenoids can be cleaved by CMOI to yield retinaldehyde.

Based on their chemical structure, carotenoids can be classified as carotenes and xanthophylls. Carotenes (like β-carotene, α-carotene and β-cryptoxanthin) are non-oxygenated carotenoids that may be linear or possess cyclic hydrocarbons at one or both ends of the molecule. Xanthophylls (like lutein, zeaxanthin, meso-zeaxanthin, astaxanthin and canthaxanthin) are oxygenated derivatives of carotenes [13,17]. Some of the carotenoids also serve as precursors of vitamin A, thus allowing their classification in provitamin A and non-provitamin A carotenoids. Provitamin A carotenoids yield vitamin A and its metabolites (retinoids) upon enzymatic and non-enzymatic cleavage, with β-carotene being the most abundant and well-characterized precursor of vitamin A in the human diet [1,18]. β-carotene contains 40 carbons with 15 conjugated double bonds and 2 β-ionone rings at both ends of the molecule [1,19,20] (Figure 1). These structural properties make β-carotene highly hydrophobic and non-polar in nature. Overall, all carotenoids are highly hydrophobic molecules.

## 3. β-Carotene as a Source of Retinoids

Out of the several hundred carotenoids that have been identified in nature only a few are abundantly present in the human diet and detected in human bloodstream and tissues. These are α-carotene, β-carotene and β-cryptoxanthin (provitamin A carotenoids); and lycopene, lutein, zeaxanthin and meso-zeaxanthin (non-provitamin A carotenoids) [13].

About 30% of the dietary vitamin A intake in western countries is contributed by β-carotene, but in the developing countries it represents the most abundant, and in some instances the sole source of vitamin A [9,21]. β-Carotene, predominantly found in nature as all-*trans* β-carotene, is the only carotenoid that can yield 2 molecules of retinaldehyde upon its symmetrical cleavage by the enzyme β-carotene-15,15′-oxygenase (CMOI or BCMO1 or BCO1) [13,22,23]. However, β-carotene can also be cleaved asymmetrically by the enzyme β-carotene-9′,10′-oxygenase (CMOII or BCDO2 or BCO2), to generate a β-ionone ring and apocarotenals, which can be ultimately converted to one molecule of retinaldehyde [13,22,23]. The mechanism of the latter conversion has not been completely elucidated (Figure 1).

Retinaldehyde formed upon the cleavage of provitamin A can be oxidized by the action of enzymes of the retinaldehyde dehydrogenase family (RALDH or ALDH 1 family) to generate all-*trans* retinoic acid, the biologically active form of vitamin A [24]. Retinoic acid acts predominantly, even though not exclusively, as a transcriptional regulator [25,26]. It functions as a ligand for specific nuclear receptors, retinoic acid receptor (RAR) or retinoid X receptor (RXR) that form homo- or hetero-dimers to regulate the transcription of several hundred target genes [25,26]. When the production of retinoic acid in tissues exceeds certain limits, enzymes that belong to the family of cytochrome P450 can carry out its oxidative degradation to generate more polar compounds, like 4-hydroxy or 4-oxo retinoic acid, which are believed to be transcriptionally inactive [27] (Figure 1).

Alternatively, retinaldehyde can be reversibly reduced to retinol [24], the alcohol form of vitamin A, which is most commonly referred to as “vitamin A” [28]. Various members of the retinol dehydrogenase family of enzymes catalyze this reaction [24]. Retinol can then be esterified mainly by the action of lecithin:retinol acyltransferase (LRAT) to generate retinyl ester, which is the storage form of vitamin A in various tissues, predominantly liver, but also lung, adipose tissues, heart and kidney [29,30,31] (Figure 1). LRAT constitutes about 90% of the catalytic activity utilized for retinyl ester formation, especially in the liver [28]. Indeed, mice lacking LRAT have no detectable retinyl ester stores in their livers [30,31]. Unlike LRAT, that uses lecithin as a fatty acid donor, another retinyl esterase utilizes acyl CoA to catalyze the formation of retinyl esters from retinol (acyl CoA:retinol acyltransferase, ARAT). Such retinyl esterase activity has been reported in rat liver microsomes [32], in the rat intestine [33], in the human intestinal Caco-2 cell line [34] and more recently in the mouse embryo [35]. However, the molecular identity of the enzyme that exhibits such activity is yet to be confirmed. The enzyme acyl CoA:diacylglycerol acyltransferase 1 (DGAT1) has been shown to function as an ARAT that esterifies retinol to retinyl esters in murine skin [36] and intestine [37], under conditions in which dietary retinol exceeds the capacity of LRAT to esterify it.

## 4. The β-Carotene Cleavage Enzymes

In humans and mice, both β-carotene cleavage enzymes (CMOI and CMOII) are expressed in various adult tissues, including liver and adipose, as well as in the developing tissues such as placenta, yolk sac and the embryo [38,39,40,41,42,43,44]. These enzymes can carry out the bioconversion of β-carotene to vitamin A *in situ*, suggesting that β-carotene can serve as a local source of retinoids at various sites within the body.

β-carotene-15,15′-oxygenase (CMOI)–CMOI is a cytosolic enzyme with a strong substrate specificity, being able to interact only with carotenoids such as β-carotene with at least one non-substituted β-ionone ring [45]. CMOI is the main enzyme that cleaves β-carotene to generate vitamin A *in vivo* in adult tissues [46,47]. Indeed, when fed a diet containing β-carotene (at a final concentration of 1 mg/g of diet) as a sole source of vitamin A for 16 weeks, mice lacking CMOI (CMOI−/−) were unable to cleave dietary β-carotene and accumulated this carotenoid in large quantities in tissues, as seen by the orange color of the adipose tissues [46]. In accordance with this effect, vitamin A levels were significantly lower in various tissues including lung, kidney, testis and uterus of the knockout mice [46]. CMOI also plays a leading role in utilization of β-carotene by the developing tissues. Our laboratory generated a mouse strain lacking CMOI in the retinol-binding protein (RBP or RBP4) knockout background [44]. RBP is the sole specific carrier of retinol in the bloodstream where it is secreted from the liver to transport the stored vitamin A towards the periphery of the body [48]. The RBP−/− strain is an established model of embryonic vitamin A-deficiency [49]. β-Carotene supplementation of the CMOI−/−RBP−/− dams, carrying embryos expressing one copy of the enzyme (CMOI+/−RBP−/−), ameliorated the features of vitamin A deficiency of these embryos [44]. This study unequivocally confirmed that intact β-carotene can be taken up from the maternal circulation by the developing tissues and can be cleaved *in situ* by the action of embryonic CMOI to synthesize retinoids locally [44].

In the past few years, a role of CMOI in regulating lipid homeostasis has been proposed. This potential action is based on the observations that regardless of the vitamin A content of the diet, CMOI−/− mice accumulate lipids in the serum and liver, show altered hepatic expression of genes involved in fatty acid metabolism, and show increased mRNA levels of PPARγ-activated genes in visceral adipose tissue [46,50,51]. In addition, CMOI−/− mice are more susceptible to diet-induced obesity and develop a severe fatty liver phenotype accompanied by increased levels of serum free fatty acids and cholesteryl esters when maintained on a high-fat diet [46,50,51]. Further *in vitro* studies supported the hypothesis that retinoids, such as retinal and retinoic acid, formed upon the cleavage of β-carotene by CMOI may influence lipid metabolism in adipocytes by modulating PPARγ and RAR signaling pathways [50,51]. Nevertheless, it is still not clear whether CMOI affects lipid metabolism in various tissues in a similar manner and whether such action is independent of its ability to cleave β-carotene. Overall, the molecular mechanism underlying the proposed action of CMOI to modulate lipid metabolism has not been fully elucidated. In this regard, our laboratory has suggested that CMOI may perform alternative functions in addition to generating retinoids from β-carotene, at least during embryogenesis. We first showed that lack of this enzyme in the embryo led to reduced LRAT mRNA expression and activity, thus impairing retinyl ester formation [44]. More recently, we reported that embryonic CMOI influences the formation of fatty acyl esters derived not only from retinol, but also from cholesterol and diacylglycerols [35]. Long chain unsaturated fatty acid moieties of several lipid sub-classes were severely attenuated in the absence of embryonic CMOI. This observation points to an important and novel role of this enzyme in the homeostasis of specific lipids that are crucial for embryonic development, likely for the developing nervous system, where long chain polyunsaturated fatty acids are highly concentrated [52,53,54]. These data add an additional layer of complexity to the alternative function(s) that CMOI may play in the developing tissues and may provide additional clues to understand the cross-talk between lipid and carotenoid metabolism.

It remains to be elucidated whether this action of CMOI is restricted to the embryo or also takes place in adult tissues. Furthermore, it is important to understand the molecular mechanisms underlying this novel function of the symmetric cleavage enzyme. Based on several experimental findings, we proposed that multiple mechanisms could be involved. On the one hand, CMOI may influence the transcription of certain genes and may in turn affect the enzymatic activities of their corresponding proteins that catalyze the synthesis of retinyl ester, cholesteryl ester and triacylglycerols. Such candidate genes are *LRAT*, lecithin:cholesterol acyltransferase (*LCAT*), acyl CoA:cholesterol acyltransferase 1 (*ACAT1*) and diacylglycerol O-acyltransferase 2 (*DGAT2*) [29,55,56,57]. On the other hand, CMOI may be more directly involved in these acyltransferase reactions acting, for example, as a lipid transporter. Overall, further studies are needed to elucidate the alternative function(s) of this enzyme in detail and its potential impact on human health.

β-carotene-9′,10′-oxygenase (CMOII)–CMOII is a second mammalian carotenoid cleavage enzyme that was first characterized as being able to asymmetrically cleave β-carotene *in vitro* and to cleave non-cyclic carotenoids like lycopene, both *in vitro* and *in vivo* [58,59]. Later, it was shown that CMOII has much broader substrate specificity with a higher preference to cleave carotenoids with 3-hydro-ionone rings, like xanthophylls lutein and zeaxanthin, and canthaxanthin with 4-oxo-substituted ring sites [60,61]. The contribution of the asymmetric cleavage by CMOII to generate vitamin A *in vivo* is thought to be minor compared to that of CMOI [22,60]. In the section below, we will discuss novel evidence in regards to this action recently reported by the von Lintig group [45].

CMOII has recently been shown to behave like an oxidative stress-regulated protein that protects mitochondria from the carotenoid-activated apoptotic cascade [60,61]. Therefore, CMOII seems to act as a carotenoid scavenger and a gatekeeper of apoptotic pathway in mitochondria, which is the site of its subcellular localization [60,61]. Data in the literature indicate that such scavenging action of CMOII could also be directed towards the synthetic retinoids like 4-oxo-fenretinide (4-oxo-4HPR) [61]. Whether other retinoids could be similarly scavenged remains to be addressed. We find the work by Hammerling and colleagues intriguing in this regard. These researchers have identified retinol as a crucial component of the mitochondrial PKCδ signalosome [62,63,64]. In its activated state, PKCδ signals to the pyruvate dehydrogenase complex for enhanced production of acetyl CoA from pyruvate, thus increasing both respiration and ATP synthesis. Within the signalosome, vitamin A acts as a co-factor for redox activation of PKCδ by functioning as an electron carrier, similar to ubiquinone in the electron transfer chain, by virtue of its conjugated double bond system [62,63,64]. Hammerling and colleagues proposed that handling electrons by this highly adaptable system of conjugated double bonds provides the central chemical feature underlying the physiological properties of retinoids and carotenes. Given these findings and the mitochondrial localization of CMOII, we wonder whether by scavenging carotenoids and/or other retinoids, CMOII can also participate in maintaining the function of the mitochondrial PKCδ signalosome and if so, what would be the impact of this action on human health. 

β-Apocarotenoids–it is well established, that in addition to the symmetric cleavage by CMOI, enzymatic and non-enzymatic cleavage of β-carotene at non-central double bonds can also occur [13]. The products of these reactions are β-apocarotenals and β-apocarotenones, whose biological functions in mammals have only begun to being elucidated. Shmarakov and colleagues reported the detection of various β-apocarotenals, including apo-8′-, apo-10′-, apo-12′-, and apo-14′-carotenal, in a mouse diet formulated using β-carotene beadlets and in the beadlets themselves [65]. It is now confirmed that β-carotene-containing animal diets and any dietary source of β-carotene also contains β-apocarotenoids [6]. Interestingly, Harrison and colleagues [66] demonstrated that β-apo-14′-carotenal, β-apo-14′-carotenoic acid, and β-apo-13-carotenone antagonized all-*trans* retinoic acid-induced transactivation of all three RARs, at nM concentrations, likely by directly competing with retinoic acid for high affinity binding to purified receptors. Finally, these β-apocarotenoids inhibited the retinoic acid-induced expression of retinoid responsive genes in HepG2 cells [66]. In a previous study, the same authors also showed that β-apo-13-carotenone, β-apo-14′-carotenal and β-apo-8′-carotenal antagonized the activation of RXRα by 9-*cis* retinoic acid, with various degrees of potency [67]. Moreover, Ziouzenkova and colleagues demonstrated that β-apo-14′-carotenal inhibited agonist-induced RXRα, PPARα and PPARγ activation very effectively and that this β-carotene metabolite decreased adipogenesis in a concentration dependent manner by regulating the expression of genes that are known targets of PPARγ in 3T3-L1 cells [68]. Together these data strongly support the notion that specific β-apocarotenoids function as antagonists of nuclear receptors and specifically exert an anti-vitamin A activity. It has been proposed that this latter action of the β-apocarotenoids could be responsible of the detrimental effects of high doses (30 mg/day) of β-carotene in human clinical trials of cancer prevention, such as the CARET trial, which had to be stopped early due to the increased incidence of lung cancer in the supplemented smoker subjects [11]. Similarly, the ATBC study showed that pharmacological doses of β-carotene (20 mg/day) in combination with alpha-tocopherol (50 mg/day) did not prevent lung cancer in older heavy smoker men but rather increased lung cancer incidence in these subjects [10]. In both cases, the high dose of β-carotene, coupled with the increased oxidative stress of smoking, would lead to enhanced eccentric cleavage of β-carotene thus generating a mixture of cleavage products that would disrupt the retinoid signaling [66,69].

It has been proposed that the asymmetric cleavage of β-carotene enables the production of β-apocarotenoids [70]. However, the *in vivo* contribution of CMOII to this process has not been unambiguously demonstrated or ruled out. A very recent report from von Lintig and colleagues has started to shed light on this issue [45]. By using specific knockout mice for the two β-carotene cleavage enzymes, as well as *in vitro* experiments, these authors reported that CMOII catalyzes the *in vivo* production of a specific β-apocarotenoid, apo-10-carotenol, which can be esterified by LRAT. Apo-10-carotenol in turn can trigger RBP release and can be taken up by target cells via the RBP-specific receptor STRA6 [45]. Even more interestingly, the authors showed that apo-10-carotenol can be metabolized by CMOI to be converted to retinaldehyde, and that CMOI can cleave other β-apocarotenoids as well. This latter finding confirms an earlier literature report showing that the concentration of β-apocarotenoids in serum and tissues of CMOI−/− mice fed a diet supplemented with β-carotene tended to be greater than those of wild-type mice under a similar dietary regimen [65]. While these data were consistent with the elevated levels of CMOII expression reported in the CMOI−/− liver (increased β-apocarotenals formation through eccentric cleavage), they also raised the possibility that CMOI could cleave β-apocarotenoids [65]. In another recent report, Harrison and co-workers also demonstrated that CMOI catalyzes the oxidative cleavage of β-apo-8′-carotenal to yield retinaldehyde. However, the shorter β-apocarotenals (β-apo-10′-carotenal, β-apo-12′-carotenal, β-apo-14′-carotenal) did not show Michaelis-Menten behavior under the conditions tested [71]. Von Lintig and colleagues provided evidence that CMOII alone does not significantly contribute to β-carotene homeostasis *in vivo* and suggested that it is unlikely that the asymmetric cleavage enzyme is a component of a pathway for the production of β-apocarotenoid signaling molecules that could interact with nuclear receptors [45]. In contrast, they proposed a coordinated action of CMOI and CMOII (“stepwise cleavage”) in generating retinoids from asymmetric provitamin A carotenoids such as β-cryptoxanthin [45]. These studies have paved the road to new areas of investigation. What is the relationship between the actions of the two cleavage enzymes in regards to their different subcellular localization, and to the consequent shuttling of carotenoids and their derivatives is certainly the next crucial question to answer.

## 5. Intestinal Absorption of β-Carotene and Its Plasma Levels

The small intestine is responsible for absorbing dietary lipids and lipid-soluble vitamins, including β-carotene, to subsequently deliver them to the peripheral tissues. Even though the human intestine abundantly expresses the main β-carotene cleavage enzyme CMOI, complete intestinal conversion of all of the ingested β-carotene to vitamin A practically does not occur. Indeed, about 17%–45% of the ingested β-carotene is released into the human circulation in its intact, uncleaved form [22,72,73]. A variable enzymatic activity of CMOI associated with a number of polymorphisms in the *CMOI* gene seems to be responsible for the less efficient cleavage in some individuals [74,75,76,77]. In contrast, mice and other rodents are considered very efficient cleavers of ingested β-carotene in the intestine, and only upon intake of supra-physiological quantities, this provitamin A carotenoid can be detected in their circulation [78]. Other animal models such as Mongolian gerbils [79,80], domestic ferrets [81,82,83] and pre-ruminant calves [84,85,86] also absorb dietary β-carotene in its intact form and have plasma and tissue distribution of the provitamin A similar to humans. In humans, the concentration of intact β-carotene in the plasma is a good indicator of bioavailability of ingested β-carotene [87,88], which represents the amount of the provitamin A absorbed by the intestinal epithelia that is available for the use by the body. In addition to the above-mentioned polymorphisms in the *CMOI* gene, single nucleotide polymorphisms (SNPs) in genes involved in lipid metabolism, such as apolipoprotein B (*apoB*), A-IV (*apoA-IV*), scavenger receptor B I (*SR-BI*) and lipoprotein lipase (*LPL*), have also shown to affect the plasma levels of β-carotene and individual carotenoid status [89,90,91,92,93]. The proteins encoded by these genes are likely involved in controlling transport and uptake of β-carotene. In addition to genetic factors, the bioavailability of β-carotene seems to be also affected by the nature of food matrix, fat content of the diet, type of fat, digestibility of fat-soluble components in the diet, bile acids, interactions with other carotenoids and individual variations due to endogenous activity of the digestive enzymes [94]. Further details on the mechanisms of intestinal absorption of dietary β-carotene and the various factors that influence this process to ultimately regulate β-carotene bioavailability are provided by E. Reboul in a review article of this special issue [95].

## 6. Transport of β-Carotene in the Bloodstream

It has long been established that β-carotene (like other carotenoids), being highly lipophilic and non-polar, is transported in the circulation in association with various classes of lipoproteins. It could likely be incorporated into the hydrophobic core of various lipoprotein particles such as chylomicrons and their remnants, VLDL, IDL and LDL along with other lipids such as cholesteryl esters and retinyl esters [96,97,98,99]. These lipoproteins facilitate its transport from the intestinal barrier to various tissues of the body, as well as its transfer across tissues.

Plasma response upon oral β-carotene dose was studied by Johnson and Russell in 1992 in male subjects [96]. They observed an early rise (3–6 h post consumption) in the β-carotene concentration in chylomicrons. These levels peaked at 6 h and dropped afterwards due to clearance from the circulation. β-Carotene in the VLDL fraction was elevated through 3 days post-consumption, due to hepatic re-secretion of these particles. Relatively low amounts of β-carotene were associated with IDL and the highest increase in β-carotene concentration was observed in LDL at 2–2.5 days post consumption. HDL particles also contained β-carotene at later time points [96]. The results from this study suggested that β-carotene can be incorporated into all the classes of lipoproteins to a varying degree and its incorporation at various time points indicates a dynamic exchange of this provitamin A carotenoid among various lipoproteins [96]. A similar study by Traber and colleagues [100] also showed the first appearance of the provitamin A carotenoid in chylomicrons upon oral β-carotene administration in 9 human subjects. It was followed by a rise in β-carotene concentrations in VLDL at later time intervals. β-carotene was detected in the HDL only upon chylomicron clearance up to 11 h post-consumption, whereas its concentrations in the LDL increased for up to 48 h [100]. Ribaya-Mercado and colleagues reported that upon β-carotene consumption, LDL fractions in the plasma of 10 women subjects showed the highest rise in β-carotene levels followed by a rise in HDL and VLDL fractions [101]. Overall, these findings are in agreement with earlier reports showing that about 60%–70% of intact β-carotene is transported in LDL in the human circulation [97]. In the fasting circulation, β-carotene is mainly associated with VLDL and LDL, the lipoproteins containing apoE and apoB moieties [99], and in the postprandial circulation β-carotene in the triglyceride-rich fraction, *i*.*e*., VLDL and chylomicrons, is considered a marker of intestinal β-carotene absorption [102].

Among other species, ruminants are considered a good model to study carotenoid transport as they are inefficient cleavers of intestinal carotenoids just like humans and have similar plasma and tissue distribution of carotenoids. Oral administration of β-carotene in calves followed by analysis of their plasma lipoproteins showed higher percentage of β-carotene associated with LDL [86]. Ashes *et al.* [103] reported HDL as the β-carotene carrier in the bovine circulation. We wonder what would be the distribution of β-carotene within lipoproteins in the mouse, given that this rodent has a higher prevalence of HDL in the bloodstream. Overall, studies in various mammalian species emphasize that β-carotene can be transported in association with various lipoproteins in the circulation, even though different lipoproteins may preferentially transport this provitamin A carotenoid in different species. This possibility raises one more time the issue of identifying the most appropriate model to study β-carotene transport in mammalian systems.

## 7. Tissue Uptake of β-Carotene

β-carotene can be acquired from the bloodstream by various tissues within the body, to be stored or be readily metabolized. In mammals, liver is a major organ that accumulates large quantities of β-carotene, followed by adipose tissue, kidney, skin and lung [104,105]. However, other tissues including adrenal gland, testes, and mammary gland can also store this provitamin A carotenoid [106]. In addition, β-carotene is also detected in placenta, yolk sac and embryo [44,107]. Unlike other carotenoids, such as lutein, zeaxanthin and meso-zeaxanthin that exclusively accumulate in the macular region of the retina [108,109], β-carotene has a much broader distribution within the body that correlates with the wide expression of the cleavage enzymes in various tissues. Current knowledge in regards to the mechanism of tissue uptake of β-carotene *in vivo* in mammals is still rather scarce and calls for further studies.

Since β-carotene and other carotenoids are transported in the circulation in association with lipoproteins, lipoprotein receptors are the proteins of interest that could mediate the tissue uptake of these micronutrients. The uptake of dietary β-carotene by the enterocytes has been discussed in detail by E. Reboul [95] in this special issue. Briefly, we would like to highlight that evidence indicates SR-BI, the specific receptor for HDL [110], as a key player in the intestinal absorption of β-carotene [92,111,112,113]. Intestinal uptake and β-carotene conversion into retinoids have been shown to be regulated by a feedback mechanism depending upon the vitamin A status. Indeed, both these processes are attenuated when dietary vitamin A is in excess to prevent accumulation of toxic levels of retinoids [112]. At the molecular level, this response is mediated by ISX, an intestinal specific transcription factor that negatively regulates the expression of both CMOI and SR-BI [112]. ISX expression is upregulated by retinoic acid [114,115]. Thus, when intestinal retinoic acid levels are high, as in the case of excessive intake of vitamin A, ISX levels increase to downregulate the expression of SR-BI and CMOI [112]. We refer the reader to the above-mentioned review [95] for additional information on the potential role of other key players in lipid metabolism in the intestinal uptake of β-carotene and other carotenoids. The main evidence for the involvement of such candidate proteins, including CD36, apolipoproteins B, E and CIII, LPL and ABC transporters, comes from *in vitro* studies or from human studies that have identified gene polymorphisms linked to different levels of circulating carotenoids. The molecular proofs of the unequivocal role of these other lipid transporters in modulating β-carotene uptake are still missing.

Even though it is generally recognized that the liver can take up various carotenoids, including β-carotene, to metabolize these molecules or re-secrete them in VLDL, whether the lipoprotein receptors on liver parenchymal cells such as SR-BI, low-density lipoprotein receptor (LDLr) or LDLr related protein-1 (LRP1) mediate carotenoid uptake has not been studied. In particular, LDLr aids in the endocytosis of the majority of lipoproteins in the circulation due to its high affinity to both apoB and apoE containing lipoproteins [116]. Thus, we speculate that LDLr could play an important role in the uptake of β-carotene, at least in the liver.

In a recent report from our laboratory, we provided evidence that LRP1 and possibly LPL, the enzyme that hydrolyzes triglycerides within lipoproteins, may mediate the placental uptake of intact β-carotene from the maternal circulation [107]. A single β-carotene supplementation by intraperitoneal injection at mid-gestation of wild-type dams, maintained on a regimen of copious vitamin A intake (29 IU vitamin A/g diet), induced a marked reduction of placental mRNA levels of *LRP1*. A similar effect was not observed in maternal liver, suggesting a tissue-specific response to β-carotene availability. LRP1 has high affinity for apoE-containing particles, such as VLDL, chylomicrons and their remnants, indicating that at least under this experimental condition, β-carotene may be predominantly incorporated in apoE-containing lipoproteins and that LRP1 may mediate its uptake at the placental barrier. We proposed that the down-regulation of *LRP1* mRNA expression may result in a potential feedback mechanism that prevents the placenta from acquiring excessive maternal circulating β-carotene when the dams are on a regimen of copious vitamin A intake [107]. In the same report, we also analyzed placental uptake of β-carotene in dams lacking LRAT and RBP (LRAT−/−RBP−/−) maintained on a regimen of copious vitamin A intake, as indicated above. Under the above-mentioned dietary conditions, LRAT−/−RBP−/− mice can be considered a model of marginal vitamin A deficiency due to their extremely low concentration of hepatic retinoid stores and serum retinol-RBP that make them highly susceptible to developing signs of vitamin A deficiency [117]. In this model, the enhanced accumulation of β-carotene in the placenta was accompanied by the upregulation of placental *LPL* mRNA, suggesting a potential critical role of LPL in mediating placental uptake of the provitamin A carotenoid, at least in response to a tenuous vitamin A status [107]. Intriguingly, placental LPL has already been shown to facilitate uptake of postprandial retinoids [118].

Overall, more studies are needed to elucidate the tissue-specific mechanisms of uptake and subsequent metabolism of β-carotene.

## 8. Intracellular Trafficking of β-Carotene

Very little is known in regards to how β-carotene is transported within the mammalian cells, despite the importance of this process that could influence intracellular accumulation and metabolism of this provitamin A carotenoid. Most of the current knowledge in this area pertains to the intracellular trafficking of carotenoids other than β-carotene and even in this case, many questions remain unanswered. Bernstein and colleagues have devoted considerable efforts in understanding the intracellular trafficking of lutein and zeaxanthin in the macula of the human eye where these carotenoids are highly concentrated [108,109]. Uptake of lutein and zeaxanthin circulating in the bloodstream should occur first in the retinal pigment epithelium (RPE), where SR-BI could likely be involved. Very recent data suggest that the interphotoreceptor retinoid-binding protein (IRBP), that shuttles retinoids from the RPE to the retina, can also facilitate the transfer of xanthopylls, even though the authors did not completely rule out a direct delivery of lutein and zeaxanthin to the macula via the retinal circulation [119]. In the retinal cells, CD36 could mediate the uptake of these carotenoids, but specific binding proteins for zeaxanthin (Glutathione S-transferase P1 isoform, GSTP1; [120]) and lutein (StARD3, a member of the steroidogenic acute regulatory domain -StARD- protein family; [121]) working in concert with tubulin [122,123] ultimately seem to facilitate the selective delivery and accumulation of these carotenoids within the macula. Similar mechanisms are employed by invertebrates, such as the silkworms, to deliver lutein to the silk gland where a specific cell surface uptake protein, Cameo2, and a specific carotenoid binding protein, CBP, are needed [124,125].

## 9. Conclusions

Although this year marks the century of vitamin A research, there are still various aspects of the mammalian metabolism of its main precursor, β-carotene, which are not fully understood. β-Carotene bioconversion by its cleavage enzymes has been studied extensively. Interesting recent studies have shown that the expression and activity of its main cleavage enzyme CMOI in the intestine are regulated by diet and genetics. Investigations from our laboratory revealed that in addition to its cleavage activity, this enzyme may contribute to retinyl as well as cholesteryl ester formation in mouse embryos. Whether this effect is tissue specific and whether CMOI exerts this function by a direct involvement in the esterification process or by acting as a facilitator of it are yet to be elucidated. On the other hand, the metabolic pathway of vitamin A formation by asymmetric β-carotene cleavage due to CMOII is not fully understood. In addition, β-apocarotenoids, the products of such cleavage are recently shown to modulate nuclear receptor signaling. It needs to be confirmed whether antagonistic activity of β-apocarotenoids to RXR signaling is responsible for detrimental effects of high doses of β-carotene as observed in the CARET study. It also remains to be explained whether the two β-carotene cleavage enzymes interact and whether the nature of such interaction is synergistic or discordant.

Furthermore, the mechanisms of uptake of β-carotene have been studied mainly in the intestine and SR-BI has been shown to be the mediator of such process. It is not fully known whether the uptake of intact β-carotene from the circulation in other tissues is a protein mediated process and if so, which are the key proteins involved in such process. Studies from our laboratory have suggested a role of LRP1 and LPL in mediating placental uptake of this carotenoid from the circulation, at least under certain experimental conditions. However, the molecular details of the receptor mediated β-carotene uptake need to be investigated. 

Current knowledge regarding intracellular carotenoid trafficking is limited to xanthophylls and is virtually missing in regards to β-carotene. Some studies have speculated a protein facilitated transport of β-carotene in the cells, however the existence of such a transport mechanism needs to be unequivocally confirmed. 

Filling the gaps in knowledge highlighted in this review will enable a thorough understanding of the metabolism of β-carotene in mammals and ultimately provide the appropriate tools to formulate adequate dietary recommendations that will enhance the beneficial effects and reduce the detrimental consequences of β-carotene intake throughout the life cycle.
